# Diet has independent effects on the pace and shape of aging in *Drosophila melanogaster*

**DOI:** 10.1007/s10522-017-9729-1

**Published:** 2017-09-15

**Authors:** C. Ruth Archer, Ugofilippo Basellini, John Hunt, Stephen J. Simpson, Kwang Pum Lee, Annette Baudisch

**Affiliations:** 1 Centre for Ecology and Conservation, University of Exeter, Cornwall Campus, Treliever Road, Penryn, Cornwall TR10 9FE UK; 2 MaxNetAging Research School, Max Planck Institute for Demographic Research, Konrad-Zuse-Straße 1, 18057 Rostock, Germany; 3 Institut national d’études démographiques (INED), F-75020 Paris, France; 4 School of Science and Health, Hawkesbury Institute for the Environment, Western Sydney University, Locked Bag 1797, Penrith, NSW 2751 Australia; 5 Charles Perkins Centre and School of Life and Environmental Sciences, D17, Charles Perkins Centre Research and Education Hub, The University of Sydney, Sydney, NSW 2006 Australia; 6 Department of Agricultural Biotechnology, Seoul National University, Seoul, 08826 Republic of Korea; 7 Max-Planck Odense Center on the Biodemography of Aging, Department of Public Health, University of Southern Denmark, J.B. Winsløws Vej 9B, 5000 Odense C, Denmark; 8 Department of Biology, University of Southern Denmark, Campusvej 55, 5230 Odense C, Denmark

**Keywords:** Dietary restriction, Fruit flies, Geometric framework of nutrition, Gompertz, Pace, Shape

## Abstract

Studies examining how diet affects mortality risk over age typically characterise mortality using parameters such as aging rates, which condense how much and how quickly the risk of dying changes over time into a single measure. Demographers have suggested that decoupling the tempo and the magnitude of changing mortality risk may facilitate comparative analyses of mortality trajectories, but it is unclear what biologically meaningful information this approach offers. Here, we determine how the amount and ratio of protein and carbohydrate ingested by female *Drosophila melanogaster* affects how much mortality risk increases over a time-standardised life-course (the shape of aging) and the tempo at which animals live and die (the pace of aging). We find that pace values increased as flies consumed more carbohydrate but declined with increasing protein consumption. Shape values were independent of protein intake but were lowest in flies consuming ~90 μg of carbohydrate daily. As protein intake only affected the pace of aging, varying protein intake rescaled mortality trajectories (i.e. stretched or compressed survival curves), while varying carbohydrate consumption caused deviation from temporal rescaling (i.e. changed the topography of time-standardised survival curves), by affecting pace and shape. Clearly, the pace and shape of aging may vary independently in response to dietary manipulation. This suggests that there is the potential for pace and shape to evolve independently of one another and respond to different physiological processes. Understanding the mechanisms responsible for independent variation in pace and shape, may offer insight into the factors underlying diverse mortality trajectories.

**Electronic supplementary material:**

The online version of this article (doi:10.1007/s10522-017-9729-1) contains supplementary material, which is available to authorized users.

## Introduction

In a wide range of species, what individuals eat affects how long they live, how much they reproduce and how healthy they are (Simpson and Raubenheimer 2012). Traditionally, research on the effects of nutrition on survival has focussed on Dietary Restriction (DR), where individuals are fed a restricted diet relative to fully fed animals, but not such a restricted diet that it causes malnutrition. These studies have found that restricting total energy intake can extend lifespan in species including yeast (Jiang et al. 2000), mice, rats (Simons et al. 2013; but see Swindell 2012), and rhesus monkeys (Mattison et al. 2017; but see Mattison et al. 2012). However, it is increasingly clear that it is not just how much food that individuals eat that affects their survival, the ratio of nutrients eaten also plays an important role (Simpson and Raubenheimer 2012). For example, in ants (Dussutour and Simpson 2012), crickets (Harrison et al. 2014) and fruit flies (Lee et al. 2008) lifespan peaks in individuals that eat low protein, high carbohydrate diets.

While we have a good understanding of how nutrition affects lifespan (Le Couteur et al. 2016), it is less clear how different nutrients interact to affect age-dependent mortality risk. Understanding how diet affects mortality risk across the life-course to achieve lifespan extension is important. For example, if dietary manipulation immediately reduces mortality risk, then the physiological mechanisms underlying this response are likely to show immediate changes too. This can suggest which of the many cellular and molecular changes induced by dietary manipulation are involved in lifespan extension and which are not (Partridge et al. 2005). If diets improve lifespan by suppressing or delaying age-associated rises in mortality, then the physiological changes that this dietary manipulation induces may be more likely to be involved in the aging process, than if diet affects mortality in an age independent way. This distinction suggests if dietary manipulation is a good tool for investigating the mechanistic basis of aging more generally. Studies asking how nutrition affects age-dependent mortality risk have primarily focused on the effects of DR. For example, Mair et al. (2003) found that two days after applying DR, *Drosophila* that had previously been on a full diet had the same risk of dying as flies that had been on a long-term restricted diet, showing that DR reduced age-independent mortality risk. However, there is some debate about whether DR primarily extends lifespan by reducing the baseline (i.e. age-independent) mortality rate in *Drosophila* (Magwere et al. 2004), or by reducing both the baseline mortality and the rate at which mortality rises over age (i.e. age-dependent mortality) (Nakagawa et al. 2012).

Debate about how DR affects mortality trajectories could reflect differences in statistical analyses (discussed in Simons et al. 2013), variation in responses to diet between strains of model organisms (Swindell 2012), or arise from the way that we usually characterize and compare mortality trajectories. We typically measure mortality by estimating parameters (e.g. aging rates, initial mortality) from parametric models (e.g. Gompertz, Weibull) (Pletcher 1999; Promislow and Pletcher 1999). However, these models often provide a poor fit to mortality data (Willekens 2001) and larger samples are needed to parameterise models than are often used in dietary manipulation studies (Pletcher 1999; Promislow and Pletcher 1999). Additionally, such models produce rate based parameters. For example, both parameters from the Gompertz mortality model (*μ*(*x*) = *a e* ^*bx*^) are expressed per unit time. The same unit of time, however, encompasses a larger or smaller fraction of lifetime relative to the average lifespan of an organism. Thus, among treatment groups of dietary manipulated individuals that vary substantially in lifespan, comparing aging rates across populations compounds the effects of changing the pace of life (i.e. lifespan) with changes in the shape of mortality over age. Several researchers have suggested that we should compare survivorship on a time-standardized scale (e.g. Pearl 1928; Pearl and Miner 1935; Izmaylov et al. 1993; Kowald et al. 1993; Lee and Goldstein 2003; Carnes et al. 2006; Lynch et al. 2010; Baudisch 2011) and this approach has been adopted by many recent aging studies (Lynch et al. 2010; Baudisch 2011; Baudisch et al. 2013; Jones et al. 2014; Bansal et al. 2015).

Proponents of time-standardized analyses of mortality trajectories suggest that this approach can provide novel insights. For example, a recent study in *Caenorhabditis elegans* tested how several treatments, including dietary manipulation, affected the shape of survival curves while controlling for variation in chronological time. The majority of treatments that altered lifespan simply affected the tempo at which worms lived and died. That is, survival curves were stretched or compressed, but their topography was unchanged (Stroustrup et al. 2016). Stroustrup et al. (2016) suggested that this illustrates that each intervention altered all physiological processes that determine the risk of death to the same extent, throughout adult lifespan. In other words, age-associated changes in physiology that affect mortality risk simply occur at a faster or slower tempo in manipulated and control animals. It seems surprising that a wide range of interventions affects the risk of every physiological driver of mortality risk in synchrony and to the same degree. However, three manipulations caused some deviation in the time-standardised shape of survival curves in this study (Stroustrup et al. 2016). This result is more in keeping with human data, where changes in lifespan do not show simple proportional rescaling. For example, in Denmark, France, Sweden, Switzerland and Japan the risk of dying when people reach 100 years fell for many years, leading to an increase in centenarians. However, this decline in the risk of dying at 100 has slowed down or stagnated in all of these countries (apart from Denmark) and although the modal increase in lifespan has increased, variation in lifespan is declining (Robine and Cubaynes 2017). This supports the idea that the rise of human life-expectancies is resulting in compression and shifting of mortality to higher and higher ages (Canudas-Romo 2008).

While there are calls to adopt time-standardised analyses of mortality data, it is not yet clear if characterising the magnitude of changing mortality risk over a time-standardised life-course tells us something biologically meaningful about aging and it is hard to build this understanding, when comparatively few studies have adopted this approach. A sensible first step in determining what time-standardised analyses of mortality tell us about the underlying physiology of aging, is determining if the tempo and magnitude of changing mortality risk over age can vary independently across, and within species. If they can, then these parameters have the potential to evolve independently and there may be merit in trying to understand what physiological processes drive this divergence. Between species, theoretical work and comparative analyses suggest the pace and the shape of mortality have evolved independently: both long and short lived species may experience a severe, mild or no increase in mortality risk over age and even declining mortality is possible at long or short lifespan (Baudisch 2011; Jones et al. 2014). While it is clear that the tempo and magnitude of age-dependent changes in mortality can vary independently of one another both within (*C. elegans*—Stroustrup et al. 2016) and between species ﻿(Baudisch 2011; Jones et al. 2014), it is unclear how often this is the case.

To improve our understanding of how diet affects age-dependent mortality risk we quantify the pace and shape of aging (Baudisch 2011) in *Drosophila melanogaster* fed diets made according to the Geometric Framework of Nutrition (GF) (Simpson and Raubenheimer 2012). The GF allows us to decouple the effects of eating different amounts and ratios of nutrients on the expression of traits of interest. We quantify pace and shape in 1008 female *D. melanogaster* fed 28 different diets, which differed systematically in their ratio and amount of protein to carbohydrate (Lee et al. 2008). These data have previously been analysed using rate based measures of mortality but here we decouple dietary effects on the pace and shape of aging to see if, like nematodes, life extending manipulations tend to rescale the tempo at which individuals live and die. We find that diet has specific and independent effects on the pace and the shape of aging, suggesting that in *D. melanogaster* specific life-extending manipulations can have independent effects on how much and how quickly mortality risk changes over age.

## Materials and methods

### Data

We calculate the pace and shape of mortality using data collected by Lee et al. (2008). In brief, 1008 female individually housed Wild-type Canton-S flies *Drosophila melanogaster* were fed one of 28 different diets made according to the GF (Simpson and Raubenheimer 2012). These liquid diets varied in their ratio and amount of protein (P) and carbohydrate (C) (i.e. P:C and P + C). Diets varied along 7 P:C ratios (0:1, 1:16, 1:8, 1:4, 1:2, 1:1, and 1.9:1) and were diluted such that the total protein (derived exclusively from yeast) and carbohydrate (derived from sucrose and partially from yeast) concentrations were 45, 90, 180, or 360 g l^−1^. Intake of each diet was accurately measured for each fly over its lifetime. To measure consumption, flies were given liquid diet in 5-μl microcapillary tubes and consumption was measured using a scale bar. Lifespan and female egg production were also measured in these flies. It is worth noting that female egg production was measured in flies that were mated only once at the beginning of the experiment, and this regime can lead to sperm limitation in some *Drosophila* species (Taylor et al. 2008). This may have resulted in reduced egg laying rates, which can in turn, increase lifespan (Partridge et al. 1987). However, the results collected by Lee et al. (2008) are almost identical to those collected by Jensen et al. (2015) where flies were mated multiply across their lives and by Reddiex et al. (2013) where fertility was assayed in females immediately post mating. In all three studies, the P:C ratio that promoted the greatest rate of female egg laying was 1:2 P:C and, in the studies that also measured lifespan, females lived longest on a P:C ratio of 1:16. Therefore, while the flies in Lee et al. (2008) may have been sperm limited, this did not shift the relationship between protein, carbohydrate, fertility and lifespan. For full details of these methods see Lee et al. (2008).

### Measuring the pace and shape of mortality

A conceptual framework to compare patterns of aging across species with vastly different life spans is the *pace shape* framework (Baudisch 2011; Wrycza and Baudisch 2014; Wrycza et al. 2015). Pace captures the tempo at which animals live and die and characterizes the time aspect of aging. Shape captures how the risk of dying changes over the life-course, standardized for time. Shape thus describes whether changes in mortality can be characterized as mild, gradual or sharp relative to its average level, and whether mortality increases (‘senescence’), decreases (‘negative senescence’) or remains constant over age (‘negligible senescence’).

As opposed to rate based approaches, which confound the pace and shape aspects of mortality (Gompertz, Weibull), and unlike scale free approaches that have been proposed (e.g. Eakin and Witten 1995), the pace shape framework characterizes the two aspects of aging with easily interpretable, non-parametric scalar values that describe how long organisms live (pace) and how much they age (shape). Several measures may be used to quantify pace and shape values, but not all are equally intuitively appealing or theoretically desirable. Wrycza and Baudisch (2014) and Wrycza et al. (2015) derive well-justified mathematical conditions to evaluate alternative measures and identify mean lifespan and a variant of the Gini coefficient as most appropriate measures to quantify pace and shape values respectively.

Among the many closely related quantities that characterize lifespan (i.e. the pace of life), such as life expectancy, modal age at death, quantile measures of lifespan and maximum lifespan, mean lifespan (i.e. life expectancy at age zero, which is often birth but may be defined as age at maturity) is most commonly used (Preston et al. 2001). Following Wrycza and Baudisch (2014), this is our measure of pace and is calculated as follows. Let *l*(*x*) denote the survival function, which gives the probability of surviving from age zero up to age *x*, and *f*(*x*) be the probability density function describing the distribution of deaths in the population. Life expectancy at birth captures the pace value and is defined as the average lifespan in the population, and can be computed as:

$$e(0) = \int\limits_{0}^{\infty } {l(x)dx} = \int\limits_{0}^{\infty } {xf(x)dx}$$

However, because dealing with a small sample data, we computed *e*(0) using the discrete formula, where the integral is approximated with a sum.

We use the Gini coefficient to capture shape (Wrycza et al. 2015).The Gini coefficient belongs to a group of classical statistical measures that quantify lifespan variability, including Keyfitz’ entropy, the coefficient of variation, the interquartile range, variance and the standard deviation in lifespan, Theil’s entropy index, and the mean logarithmic deviation (for a detailed description of these measures see Wilmoth and Horiuchi 1999; Van Raalte and Caswell 2013). The Gini coefficient is one of the most widely used statistical indexes to measure concentration of the distribution of a positive random variable. It is widely employed in economics to measure wealth or income inequality (Gini 1912, 1914), but it has also been used in the demographic literature to evaluate lifespan inequality (i.e. the inequality in survival within or between populations, see Hanada 1983; Shkolnikov et al. 2003). The Gini coefficient ranges between zero (case of perfect equality) and one (case of perfect inequality). In the context of lifespans in a population, the index equals zero when all the individuals die at the same age; it is large if a small group of individuals lives much longer than the rest of the population; and it equals one if all people but one die at birth and the only individual dies at a positive age (Gigliarano et al. 2017). The Gini coefficient can be expressed, among other equivalent options, in terms of the Lorenz curve (Lorenz 1905). This curve associates the cumulative proportion of survival time in a population with the cumulative proportion of individuals that receives this survival time. Analytically, the Gini index is the area between the Lorenz curve and the equality diagonal (45-degree line), divided by the whole area below the diagonal, as shown in Fig. S1.

Shape values were calculated in R (R Core Development Team 2017) using the Gini function in the “*ineq”* package (Zeileis 2015), which does not require the calculation of a continuous survival function. Code for calculating this parameter is provided in the Supplementary Information. For comparative empirical analyses of shape values, Wrycza et al. (2015) suggests using a rescaled version of the most appropriate shape measure *S* (see Wrycza et al. (2015)—Appendix S2), denoted here as *S*_*r*_, which can be interpreted as a ratio of average mortality:

$$S_{r} = \frac{1 + S}{1 - S} = \frac{1 - G}{G}$$

where S = 1 − 2*G is the non-rescaled shape measure, *G* denotes the Gini coefficient and *S*_*r*_ takes on values between [0,∞]. Negligible senescence (constant mortality over age) corresponds to values of G = 0.5 and thus S = 1. Positive senescence (increasing mortality over age) corresponds to values of G < 0.5 and S > 1. Negative senescence (decreasing mortality over age) corresponds to values of G > 0.5 and S < 1. G = 0 corresponds to the extreme case of sharp positive senescence, where everyone shares exactly the same lifespan and dies simultaneously, such that mortality rises from zero to infinity at the age at death.

### Statistical analyses

To visualise the effects of protein and carbohydrate on pace and shape values, we first created nutrient landscapes. To do this, we regressed pace and shape values onto average intake of protein and carbohydrate for each dietary treatment. The resulting nutrient landscapes closely resemble maps, where intake of each nutrient replaces latitude and longitude and peaks for a trait (high trait values) and troughs (low trait values) are visible in this landscape. Non-parametric thin-plate splines were used to produce these nutritional landscapes using the Tps function in the “FIELDS” (Fields Development Team 2006) package of R. We used the value of the smoothing parameters (λ) that minimized the generalized cross-validation score when fitting each nutritional landscape (Green and Silverman 1993). Prior to analyses, we standardized response variables to a mean of zero and standard deviation of 1 using a Z-transformation. This is because if comparing values that differ in scale, differences in nutritional landscapes may arise simply because response variables are measured in different units. All analyses were conducted on these standardized variables but nutritional landscapes were plotted for raw values to ease interpretation.

We use response surface methodologies to analyse how protein and carbohydrate intake affects the pace and the shape of aging (Lande and Arnold 1983). In brief, this approach involves building increasingly complex models by adding explanatory variables and seeing if each new term significantly improves model fit. The first model measures how trait expression increases linearly with greater intake of each nutrient independently (the linear effects of protein and carbohydrate). The next models include non-linear terms which quantify how protein and carbohydrate interact to affect trait expression (correlational effects) and quadratic effects on trait expression (i.e. peaks or troughs in trait values as a factor of protein or carbohydrate intake). After adding each new term sequentially, we compared the full and reduced model to determine if the extra terms improved model fit.

In detail, this approach involves first fitting following linear multiple regression to estimate the linear gradients for protein and carbohydrate intake on pace and shape:

1 $$R = \alpha +\varvec{\beta}P +\varvec{\beta}C + \varepsilon$$

where *R* is the response variable, **α** is the regression intercept, $\varvec{\beta}$ **s** represent the partial regression gradients for protein (*P*) and carbohydrate (*C*) and **ɛ** is the random error component. Please note that $\varvec{\beta}$P and $\varvec{\beta}$C can take on different values.

To estimate the nonlinear (i.e. quadratic and correlational) gradients for nutrient intake on the response variables, the following nonlinear multiple regression model was fitted:

2 $$R = \alpha +\varvec{\beta}P +\varvec{\beta}C + \gamma P^{2} + \gamma C^{2} + \gamma PC + \varepsilon$$

where R, **α,** $\varvec{\beta}$P and $\varvec{\beta}$C are as described for Eq. 1, and **γ** *P* ^*2*^ and **γ** *C* ^*2*^ represent the quadratic gradients for protein and carbohydrate respectively and **γ** *PC* represents the correlational gradient for these two macronutrients. Once more, **γ** *P* ^*2*^ and **γ** *C* ^*2*^ can vary. For the quadratic gradients, a negative term indicates a peak on the nutritional landscape, whereas a positive term indicates a trough. The linear terms are included but not interpreted from Eq. 2: they are simply included so that the nonlinear effects can be examined when the linear effects have been removed.

We statistically compared nutritional landscapes using a sequential model building approach (Draper and John 1988) based on partial *F*-tests (Bowerman and O’Connell 1990). In instances where an overall significant difference was detected, univariate analyses were used to determine which nutrient (P or C) contributed to this effect. For full details of this methodology, see Bunning et al. (2015).

This sequential model approach only compares the sign and magnitude of nutritional gradients and does not provide information on directionality. That is, traits may be optimised in the same region of the nutrient landscape but show differences in the sequential model because they are more or less sensitive to the intake of a particular nutrient and so trait values increase more steeply with nutrient intake. To see where peaks are in different regions of the nutrient landscape we used trigonometry to calculate the angle ($\varvec{\theta}$) between the linear nutritional vectors of the landscapes being compared as:

$$\varvec{\theta}= \cos^{ - 1} \left( {\frac{a \cdot b}{\left\| a \right\|\left\| b \right\|}} \right)$$

where *a* is the linear vector of protein and carbohydrate intake of the first response variable being compared, *b* is the linear vector of these nutrients for the second response variable, where $\left\| a \right\| = \sqrt {a \cdot a}$ and $\left\| b \right\| = \sqrt {b \cdot b}$. If $\varvec{\theta}$ = 0°, the nutritional vectors are perfectly aligned and the optima for the two response variables therefore reside in the same location in nutrient space. In contrast, $\varvec{\theta}$ = 180° represents the maximum possible divergence between vectors and indicates that the nutritional optima for the response variables being compared occupy different regions in nutritional space. To determine the significance of $\varvec{\theta}$, we estimated the 95% credible interval (CI) of this angle using a Bayesian approach implemented in the ‘MCMCglmm’ package of R (Hadfield 2010). This approach has been described in detail elsewhere (Bunning et al. 2015).

## Results

**Fig. 1:**
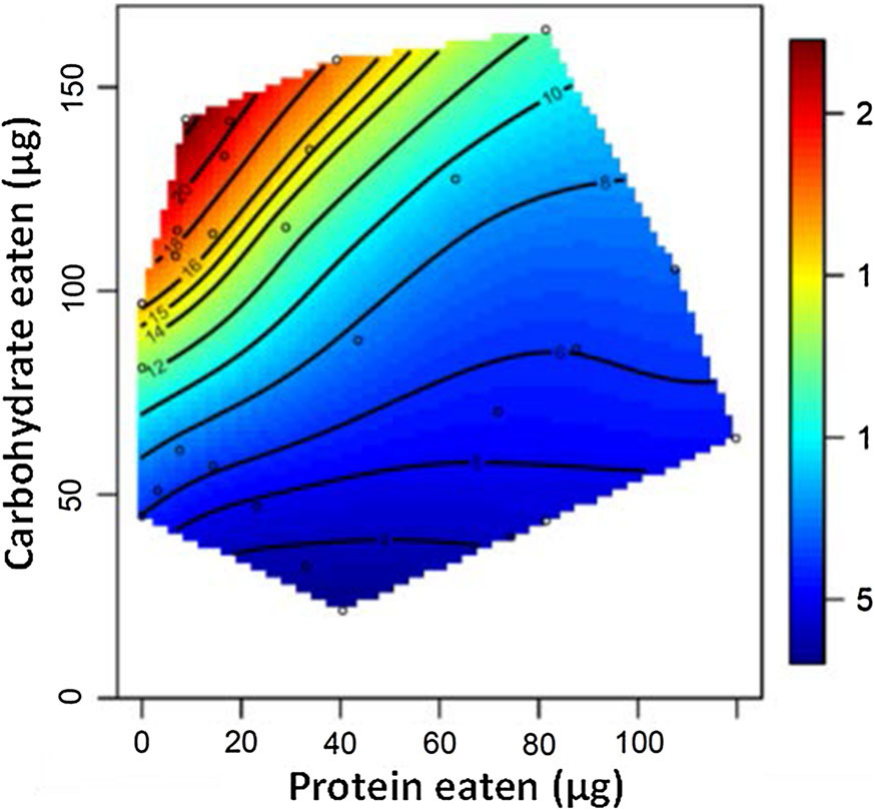
Nutritional landscapes illustrating the effects of daily protein (P) and carbohydrate (C) intake on the expression on our pace measure (mean lifespan—days). High values of these traits are given in red and low values in blue

The pace of mortality was significantly influenced by the linear gradient of both protein and carbohydrate intake. Specifically, pace values increased as flies consumed more carbohydrate but declined with increasing protein consumption (Table 1; Fig. 1). There was also a significant positive quadratic gradient for protein intake generated because pace values reached a significant minimum in flies consuming approximately 40 μg of protein per day. Additionally, there was a significant negative correlational gradient between these nutrients. Collectively, this pattern of nutritional effects means that lifespan was greatest in flies that consumed high carbohydrate, low protein diets (Fig. 1).

**Table 1:** Effects of protein (P) and carbohydrate (C) intake on mean pace and shape values

Response variable	Linear effects		Nonlinear effects		
	P	C	P × P	C × C	P × C
Mean pace					
Coefficient ± SE	−0.40 ± 0.11	0.72 ± 0.11	0.24 ± 0.09	0.14 ± 0.09	−0.48 ± 0.09
*t*_27_	3.72	6.70	2.65	1.50	5.19
*P*	0.001	0.0001	0.02	0.15	0.0001
Mean shape					
Coefficient ± SE	0.16 ± 0.19	−0.22 ± 0.19	0.09 ± 0.22	0.56 ± 0.23	−0.20 ± 0.23
*t*_27_	0.81	1.13	0.40	2.45	0.89
*P*	0.43	0.27	0.70	0.02	0.38

* The linear regression coefficients (i.e. P and C) describe the slope (***β***_***i***_) of the relationship between intake of that nutrient and the associated response variable. The quadratic regression coefficients (i.e. P × P and C × C) describes the curvature (given by **γ**_***ii***_) of this relationship: a negative **γ**_***ii***_ indicating a convex relationship (i.e. a peak in trait expression on the nutrient landscape), while a positive term illustrates a trough on the response surface. The correlational regression coefficient (i.e. P × C) describes how the covariance between the two nutrients (**γ**_***ij***_) influences the response variable, with a negative **γ**_***ij***_ indicating that a negative covariance between nutrients increases the response variable and a positive **γ**_***ij***_ indicating that a positive covariance between nutrients increases the response variable. Full details of this approach are provided in Lande and Arnold (1983)

**Fig. 2:**
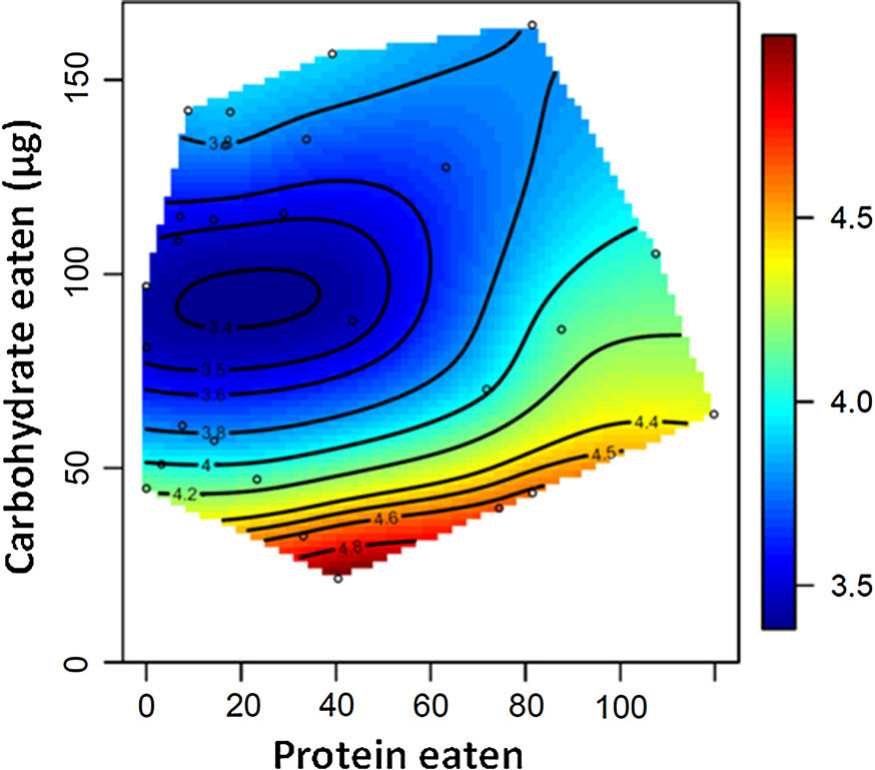
Nutritional landscapes illustrating the effects of daily protein (P) and carbohydrate (C) intake on the expression on our shape measure. High values of these traits are given in red and low values in blue

Noting that shape values greater than 1 represent an increase in mortality risk over age, while values smaller than 1 indicate a decrease over age, all our shape values indicate positive senescence. Indeed, most values are in the range of 3–4, which can be interpreted as a 3–4 fold increase of mortality over the life course, relative to average mortality. The minimum shape value is equal to about 3, which still indicates positive senescence. There were no significant linear effects of protein or carbohydrate intake on shape values. However, there was a significant quadratic gradient for carbohydrate intake indicating a trough (or minimum) for shape values in flies consuming approximately 90 μg of carbohydrate per day (Table 1, Fig. 2). This means that mortality rose less steeply in flies consuming an intermediate amount of carbohydrate but was independent of protein intake. While shape was lowest at intermediate intake of carbohydrate, this effect was not strong. There was no significant correlational effect of protein and carbohydrate intake on shape values (Table 1).

Most strikingly, when comparing how protein and carbohydrate intake affect pace and shape, it is clear that pace showed a strong response to both protein and carbohydrate and was affected by linear, quadratic and correlational terms, in contrast, shape only showed a modest response to carbohydrate intake. Accordingly, the effects of diet on pace and shape differed substantially in a number of ways. Firstly, there were significant differences in the linear effects of nutrient intake on pace and shape (Table 2). This difference was due to the fact that pace decreased with high protein intake while shape was independent of protein. Also, these linear differences reflect that pace values rose with carbohydrate intake, while shape values did not (Table 2). The angle separating the linear nutritional vectors for pace and shape was large at 140.80° (Table 2). In contrast, there was no significant difference in the quadratic or correlational effects of nutrient intake on pace and shape (Table 2). Taken together, these analyses show that the diet associated with a long life, and those associated with the least severe rise in the risk of mortality, differ significantly and do not occupy the same region of the nutritional landscape.

**Table 2:** Sequential model-building approach and angle between linear nutritional vectors for mean lifespan and mean shape

	*SS*_R_	*SS*_C_	*DF*_1_	*DF*_2_	***F***	***P***	***θ*** (95% CI)
Mean lifespan vs. mean shape							
Linear	49.59	32.76	2	50	12.84	0.0001^A^	140.80° (68.95°, 179.99°)
Quadratic	30.18	26.77	2	46	2.93	0.06	
Correlational	22.90	22.28	1	44	1.23	0.27	

*SS* sums of squares of the reduced and the complete models, *DF* degrees of freedom. Univariate tests: ^A^ P: *F*_1,50_ = 6.37, *P* = 0.02; C: *F*_1,50_ = 18.09, *P* = 0.0001

Furthermore, as protein intake varied (i.e. along the X axis in Fig. 1 and 2), the pace of life changed but shape values remained comparatively constant. This suggests that, for any given value of carbohydrate intake, changing protein intake simply rescales mortality trajectories (i.e. temporal rescaling). In contrast, moving along the carbohydrate axis in the nutritional landscapes, both shape and pace vary and do so independently; shape values are lowest when flies eat intermediate amounts of carbohydrate while pace values are lowest at very low carbohydrate intake. This pattern deviates from temporal rescaling.

## Discussion

Controlling for variation in chronological time when comparing mortality trajectories in long and short lived cohorts may help us better characterise diverse patterns of mortality (e.g. Lynch et al. 2010; Jones et al. 2014), guide research addressing the mechanistic basis of life-extending manipulations, suggest how these manipulations affect the relative duration of good versus poor health (Bansal et al. 2015) and better compare how multiple life-extending manipulations affect mortality trajectories (Stroustrup et al. 2016). Here, we apply the *pace* *shape* framework to decouple the tempo of mortality (pace) and the magnitude of changing mortality risk over a time-standardised life course (shape) to see how multiple nutrients interact to affect these two aspects of mortality. We show that pace and shape values respond independently to protein and carbohydrate intake in female *D. melanogaster.*

In female *D. melanogaster* the pace of aging is greatest in animals that consume high carbohydrate, low protein diets. In other words, high carbohydrate, low protein diets promote a long life, as clearly shown in the original analyses of these data (Lee et al. 2008). However, the diet that promotes a long life differs from the diet that promotes a less severe rise in mortality risk over the life-course. This is because the shape of aging is lowest (i.e. mortality increases least steeply over a time-standardized life-course), in animals fed intermediate amounts of carbohydrate, although variation in shape values across the nutrient landscape is modest. This result differs from previous analyses that attempted to decouple the fine-scale effects of nutrition on different mortality measures. Utilizing a modified, three-parameter version of the Gompertz mortality model Lee et al. (2008) found that the time to the onset of high mortality and aging rates peaked in similar regions of the nutrient landscape. But, both of these parameters are measured in units *time* or *1/time* respectively, highlighting their dependence on pace, and so it is not surprising that values fall in similar regions of the nutrient landscape. By decoupling the pace and shape of mortality, we can see that diet can have distinct effects on *how much* and *how quickly* cohorts senesce.

Our findings are in line with a recent study in *C. elegans.* Stroustrup et al. (2016) subjected worms to a suite of manipulations that affected lifespan and found, in general, rescaling of survival curves, i.e. constant shape values across a range of pace values. Similarly, we observe signs of temporal rescaling. As protein intake varies, while carbohydrate intake remains constant, the pace of life changes whereas shape does not. This suggests that, for any given intake of carbohydrate, varying protein intake simply rescales mortality trajectories. In contrast, we find that when moving along the carbohydrate axis in the nutritional landscapes for a given value of protein intake, both shape and pace vary and do so independently; shape values are lowest when flies eat intermediate amounts of carbohydrate, while pace values are lowest at low carbohydrate intake. This pattern therefore deviates from temporal rescaling. Hence we conclude that how mortality trajectories vary in response to dietary manipulation depends critically on which nutrient is being manipulated. Notably, Stroustrup et al. (2016) found that three experimental populations differed from this general trend: these being *C. elegans* transferred from below to above 30°C, populations with altered feeding behaviour (*eat*-2(ad1116) mutation) or a disrupted mitochondrial complex I (*nuo*-6(qm200) mutation). This shows that, even in a species where temporal scaling appears to be the norm, altered nutrition (*eat*-2(ad1116) mutants) can affect the shape of underlying mortality trajectories over a time-standardised life-course. Collectively, given that the majority of life-extending manipulations applied by Stroustrup et al. (2016) caused temporal rescaling, and the effects of diet that we see here are far greater for pace than shape, this suggests that within species variation in lifespan may frequently be due to differences in pace. However, both studies find that some manipulations that affect longevity influence both pace and shape (i.e. cause deviation form temporal rescaling), suggesting that variation in both parameters can underpin within species differences in lifespan.

From a biological perspective, how could an intervention have independent effects on the pace and shape of mortality? If pace varies but shape does not, then the underlying topography of the mortality trajectory has not changed but the rate of progression along this trajectory has. This could happen if an intervention affects every physiological factor that affects the risk of dying equally, at the same time, in all individuals (Stroustrup et al. 2016). An analogy would be watching a video in slow- motion or in fast-forward: the outcomes are ultimately the same but everything happens at a different pace. If an intervention affects the shape of mortality rather than its pace, then that intervention affects the relative spread in, but not the level of, the average age at death. Instead of merely rescaling the tempo of physiological processes, shape effects illustrate that physiological drivers that affect mortality risk respond differently, or at different times, to this intervention. Alternatively, shape effects might indicate that some members of a cohort respond differently to particular life-extending manipulations, which might be the case if responses to that interaction depend on an individual’s condition or state. More research is needed to see how often pace and shape respond differently to other empirical manipulations that affect lifespan (e.g. mild stress, temperature etc.). Understanding why some treatments appear to affect pace but not shape, while others (e.g. carbohydrate intake) affect both pace and shape, presents an interesting avenue of future research. When shape values vary independently of pace, it will be interesting to see which physiological processes mediate the changing mortality risk in response to that intervention.

When should pace and shape be considered as an analytical tool instead of rate-based mortality measures? Time-standardized mortality analyses may be appropriate when there is any substantial variation in lifespan between cohorts. This can happen within a species as a function of sex, genotype, caste (e.g. social insects), or following experimental manipulation. Pace and shape may also be useful tools where sample sizes are too small to accurately parameterize a 2 or 3 parameter mortality model or where data do not fit a particular parametric distribution. Classifying mortality curves by two scalar pace and shape values has limitations, because details of the trajectory over age are lost and pace and shape values offer only general information about how long we live and how severely we age (i.e. the steepness of changing mortality over time). But even when parameterization of mortality curves is desirable to reveal greater detail about patterns of aging in single populations, especially when parameters have clear interpretations (such as initial mortality and aging rate in the Gompertz model), pace-standardization of parameters could help unravel the shape dimension of mortality.

Our understanding of aging has changed as researchers turn their attention to non-model organisms. Contrary to what we once thought, in some species the risk of dying does not increase following sexual maturity and instead mortality risk can remain constant or even decline as organisms grow older (Jones et al. 2014). Within the same species, the tempo of age-associated changes in mortality can vary as a consequence of sex, caste or in response to empirical manipulations such as diet. We do not fully understand the evolution or mechanistic basis of this diversity. Improving our understanding of this variation relies on our being able to quantify and compare diverse mortality trajectories, and pace and shape provide one means of doing this. However, to meaningfully study the evolution of pace (i.e. lifespan) and shape (i.e. scale-free pattern of mortality), these measures need to be properly connected to biological traits. Developing such biological interpretations relies on integrating *pace* *shape* analyses alongside mechanistic analyses and in determining how manipulations with established effects on longevity and aging rates (e.g. temperature) affect these parameters. If pace and shape can vary independently within species then each trait may evolve independently. Developing an understanding of why and how this is may offer insight into additional axes of life-history evolution.

## Electronic supplementary material

Below is the link to the electronic supplementary material.

Supplementary material 1 (DOCX 62 kb)
