# Supplementary material for: Diet has independent effects on the pace and shape of aging in Drosophila melanogaster
Source: Biogerontology. 2017 Sep 15;19(1):1–12. doi: 10.1007/s10522-017-9729-1 (PMC5765211; doi:10.1007/s10522-017-9729-1)
Supplement: Supplementary file 1 — Supplementary material 1 (DOCX 62 kb) [file 10522_2017_9729_MOESM1_ESM.docx]

**SUPPLEMENTARY INFORMATION**

**Fig. S1.** The Gini concentration index and the Lorenz curve


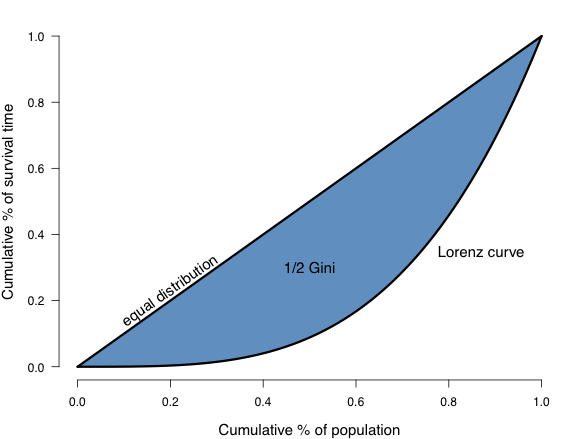


**Text S1.** R code for calculating the shape of aging

# -------- Compute rescaled shape measure from Gini coefficient -------------- #

# Notes:

# - the Gini coefficient is computed from the “ineq” package

# - different group data could be collected in columns or rows (here columns).

# All is needed to do is to choose the value of 1 (for row) or 2 (for

# columns) in the apply function (see comments in the code).

## ----------------------------------------------- ##

## -- Compute Gini and shape measures

## ----------------------------------------------- ##

## load required library

library(ineq)

## read data

mydata <- read.csv("Data.csv", header=TRUE, sep="," , na.strings = ".")

## compute the Gini coefficient (2 if columns, 1 if rows)

gini <- apply(mydata, 2, Gini, na.rm = TRUE) # here we have groups in columns

# gini <- apply(mydata, 1, Gini, na.rm = TRUE) # here we have groups in rows

## compute shape measure and its rescaled version

shape <- 1 - 2*gini

shape.rescaled <- (1-gini)/gini
